# Genetic polymorphisms in bone morphogenetic protein receptor type IA gene predisposes individuals to ossification of the posterior longitudinal ligament of the cervical spine via the smad signaling pathway

**DOI:** 10.1186/s12891-018-1966-1

**Published:** 2018-02-20

**Authors:** Hao Wang, Weitao Jin, Haibin Li

**Affiliations:** 1 Department of Orthopaedics, Beijing Tiantan Hospital, Capital Medical University, 6 TiantanXili, Dongcheng District, Beijing, 100050 China; 2 Department of Epidemiology and Biostatistics, School of Public Health, Capital Medical University, Beijing, 100069 China

**Keywords:** Ossification of the posterior longitudinal ligament, Bone morphogenetic protein receptor type IA, Single nucleotide polymorphisms, Gene transfection, Smad signaling pathway

## Abstract

**Background:**

The present study investigated the molecular mechanisms underlying the 4A > C and -349C > T single nucleotide polymorphisms (SNPs) in bone morphogenetic protein receptor type IA (*BMPR-IA*) gene, which significantly associated with the occurrence and the extent of ossification of the posterior longitudinal ligament (OPLL) in the cervical spine.

**Methods:**

The SNPs in *BMPR-IA* gene were genotyped, and the association with the occurrence and severity of OPLL were evaluated in 356 OPLL patients and 617 non-OPLL controls. In stably transfected mouse embryonic mesenchymal stem cells (C3H10T1/2), the expression levels of the *BMPR-IA* gene and Smad4 protein as well as phosphorylated Smad1/5/8 were detected by Western blotting. In addition, the alkaline phosphatase (ALP) and osteocalcin (OC) activity of osteogenesis specificity protein was assessed using the ALP quantitation and osteocalcin radioimmunoassay kit, respectively.

**Results:**

The 4A > C and the -349C > T polymorphisms of *BMPR-IA* gene were significantly associated with the development of OPLL in the cervical spine. The C allele type in 4A > C polymorphism significantly increases the occurrence and the extent of OPLL. The T allele type in -349C > T polymorphism significantly increases the susceptibility to OPLL, but not the extent of OPLL. The current results further validate our previous observations. The expression levels of *BMPR-IA* gene were significantly increased in pcDNA3.1/BMPR-IA (mutation type, MT -349C > T; MT 4A > C; MT -349C > T and 4A > C) vector-transfected C3H10T1/2 cells compared to the wild type (WT) vector-transfected cells. The levels of phosphorylated Smad1/5/8 and ALP activity were significantly increased in pcDNA3.1/BMPR-IA (MT -349C > T) vector-transfected C3H10T1/2 cells compared to the WT vector-transfected cells. However, no significant differences were observed in the protein levels of phosphorylated Smad1/5/8 and the ALP activity between MT A/C and WT vector-transfected cells. In addition, no significant differences were observed in the Smad4 protein levels among the experimental groups, as well as in the OC activity between WT vector-transfected and MT C/T, MT A/C, MT C/T and MT A/C vector-transfected cells.

**Conclusions:**

Our results suggest that Smad signaling pathway may play important roles in the pathological process of OPLL induced by SNPs in BMPR-IA gene. These results will help to clarify the molecular mechanisms underlying the SNP and gene susceptibility to OPLL.

## Background

Ossification of the posterior longitudinal ligament (OPLL) of the spine is characterized by abnormal ossification of the posterior longitudinal ligament in the spinal canal. This results in the compression of the spinal cord and nerve root, leading to serious neurological deficits as well as paralysis. Several studies have demonstrated that OPLL is a complicated multifactorial disease common in the Asian population with a prevalence of 2–4% [1]. Although a large number of genetic and environmental factors may be involved in OPLL, the etiology and pathogenesis are yet to be deciphered. Previous studies have shown that genetic factors play a crucial role in the development of OPLL [2–4]. The single nucleotide polymorphisms (SNPs) of bone morphogenetic protein (BMP) 2, 4, and 9, collagen 6A1, angiotensin I converting enzyme gene, BH3 interacting domain death agonist, nucleotide pyrophosphatase, and vitamin K epoxide reductase complex subunit 1 are significantly associated with the occurrence and development of OPLL [4–9]. These studies also suggested that the SNPs of the candidate genes increase the incidence of OPLL of an individual in an environmental specific manner. However, the molecular mechanisms underlying the activity of these susceptible genetic polymorphisms are unclear.

The bone morphogenetic protein receptors (BMPRs) are a family of transmembrane serine/threonine kinases, including three type I receptors (*BMPR-IA*, BMPR-IB, ActR-IA) and three type II receptors (BMPR-II, ActR-II, ActR-IIB) [10]. The receptor-ligand BMPs belong to the transforming growth factor-beta superfamily, and play significant roles in bone and cartilage development. Furthermore, BMP signaling is mediated by type I and type II BMP receptors. Although both types of BMP receptors are indispensable for signal transduction, the type I receptors are the high-affinity binding receptors [11]. The substrates of type I BMP receptors including the Smad protein family play a central role in BMPs signal transduction via the Smad signaling pathway. Activation of the Smad pathway leads to the expression of osteoblast-specific proteins including alkaline phosphatase (ALP), osteocalcin (OC) and collagen type I (COL1) [12–14].

Several pieces of evidence have demonstrated that *BMPR-IA* gene is a subtype of type I BMP receptors and responsible for the initiation of osteogenic differentiation [15–17]. Previous studies using immunohistochemistry staining and RT-PCR analysis have demonstrated that the expression of *BMPR-IA* mRNA and protein was elevated in the ossified ligaments of OPLL patients compared with controls. *BMPR-IA* is highly expressed in chondrocytes at the fibrocartilage tissue around the calcified zone and in fibroblast-like spindle cells at non-ossified ligaments. However, in non-OPLL patients, BMPR-IA is not expressed in the posterior longitudinal ligaments [18, 19]. In the absence of BMP2 expression, the fibroblast osteoblast activity of *BMPR-IA* gene overexpression was significantly enhanced compared to normal fibroblasts [20]. These results suggested that *BMPR-IA* gene plays an important role in the pathological ossification of OPLL. In our previous study, we demonstrated that the 4A > C and the -349C > T polymorphisms of *BMPR-IA* gene were significantly associated with the development of OPLL in the cervical spine in a Chinese Han cohort [21]. However, the molecular mechanisms underlying the 4A > C and -349C > T polymorphisms in *BMPR-IA* gene have not yet been fully deciphered. Therefore, the present study aimed to investigate the molecular mechanisms underlying the two SNPs in *BMPR-IA* gene and whether the Smad signaling pathway may be involved in the development of SNPs- induced OPLL in the cervical spine.

## Methods

### Subjects and disease criteria

The study protocol was approved by the Institutional Review Board (IRB) of Beijing Tiantan Hospital Capital Medical University. Informed consent was obtained from all the participants before the study. Study participants were recruited between January 2011 and January 2016, and consisted of 356 patients with OPLL and 617 control subjects without OPLL satisfying the inclusion criteria. The authors had access to information that could identify individual participants during or after data collection. All 973 participants were of the Han Chinese from the Beijing Tiantan Hospital Capital Medical University and resided in the northern region of mainland, China. The average age of the patients that included 199 males and 157 females was 55 years old. The control participants were age and gender matched (346 males and 271 females, 56% vs 44%). The case-control subjects were genetically homogenous. The diagnosis of OPLL was based on the criteria reported by Tsuyama [22]. Of the 356 patients with cervical spine OPLL, 131 were diagnosed as continuous type, 75 with mixed type, 118 with segmental type, and 32 with localized type. The ossification extent of OPLL was determined by the number of ossified cervical vertebrae based on lateral radiograph films. The study excluded participants with bone fluorosis, diffuse idiopathic skeletal hyperostosis, ankylosing spondylitis, and other bone metabolism diseases associated with OPLL.

### Genotyping and SNPs in BMPR-IA gene

Genomic DNA was isolated from peripheral blood of participants using the Wizard Genomic DNA Purification Kit(Promega, Madison, WI, USA). The complete coding sequence of human BMPR-IA gene (GenBank Accession No: NM004329.2) was amplified by polymerase chain reaction (PCR) using a standard protocol [23]. The DNA fragments containing the exon sequences of BMPR-IA gene was then respectively amplified using ten pairs of specific primers (Table 1). The PCR products were analyzed by direct sequencing using BigDye Terminator cycle sequencing on an ABI 3730XL POP7 DNA sequencing analysis 5.2 (Applied Biosystems, Carlsbad, CA, USA).

**Table 1:** Ten pairs of primers were used to amplify the complete exon sequences of human *BMPR-IA* gene by PCR using a standard protocol

Primer	Forward	Reverse	Annealing temperature (°C)
Primer1	GCGTTGGATGGGAGCGATAA	GGAAGCTGCGCACAGTGTTG	58
Primer2	CTCACGTCGGTCCTGTCC	CCCTGCTCCATGCCTCAC	61
Primer3	CGGAGGAGTTTATCACCTCAGCAGAGC	TTCCATCATGGCCAAAAGTTACTAGCA	60
Primer4	AGAGATTGGAATCCGCCTGCCGGGCTT	ACCCGCGAGTGGGAGACAAAAGAGG	55
Primer5	GGACTATTGAGATTGTTTAATATAC	GAAAGAACAGAAGCAAGAAATAGTG	55
Primer6	GGACTATTGAGATTGTTTAATATAC	GAAAGAACAGAAGCAAGAAATAGTG	55
Primer7	CCACAATGCATCTGGCCCCAAGGAG	TGTGATTATTACACATGGCATGCCTGTATC	55
Primer8	ACATCAGATTACTGGGAGCC	TATAGCAAAGCAGCTGGAG	52
Primer9	AAGCCTTAAGAAGATAAATGA	ATTACCCTAATGAAGTTTTTG	62
Primer10	TGATTAGTGTCTCCAGTCAAGC	TCTCAGGTAAAAGGCAAAGTC	50

### Construction of *BMPR-IA* gene expression plasmids

The single-stranded (ss) DNA oligos of human *BMPR-IA* gene (wild type, WT) encoding sequence was synthesized by BioSia, Co., Ltd., Shanghai, China. Equivalent amounts of each pair of ss DNA oligos were annealed to generate double-stranded (ds) oligos. The ds-oligos were inserted into the pGEM-T vector (Invitrogen, Carlsbad, CA, USA) using T4 DNA ligase. The construct was transformed into chemically competent *E.coli* DH5α (Invitrogen). The pGEM-T/BMPR-IA (WT) plasmid was amplified and digested with Hind III/Bam HI enzyme. The BMPR-IA cDNA (1599 bp) and the pGEM-T vector (3000 bp) were found in all the PCR products as analyzed by 4% agarose gel electrophoresis and directly sequencing, respectively. Then, the BMPR-IA cDNA (1599 bp) was inserted into the pcDNA3.1 vector (Invitrogen) using T4 DNA ligase, and the construct was transformed into DH5α cells. Using pcDNA3.1/BMPR-IA (WT) plasmid as the template, the *BMPR-IA* gene of the mutation type (MT) was amplified by PCR, using four pairs of primers (Table 2) according to the Site-Directed Mutagenesis Kit (Invitrogen). The PCR products were cloned into the pcDNA3.1 (+) plasmid. The pcDNA3.1 plasmid with the target gene and the transformants were verified by PCR and direct sequencing. The recombinant plasmid was transformed into the DH5α strain. The positive clones were designated as pcDNA3.1/BMPR-IA (WT) plasmid, pcDNA3.1/BMPR-IA (MT -349C > T) plasmid, pcDNA3.1/BMPRIA (MT 4A > C) plasmid, and pcDNA3.1/BMPR-IA (MT -349C > T and 4A > C) plasmid, respectively.

**Table 2:** Four pairs of primers were used to amplify and synthesize the human *BMPR-IA* gene for the following mutations (MT -349C > T, MT 4A > C) by PCR using pcDNA3.1/BMPR-IA wild type plasmid as the template

SNPs	Primer	Forward	Reverse	Annealing Temperature (°C)
-349C > T	Primer 1	CCGGAATTCACCATGGTGGCCGGGACC	CCGACGCCGCCGCCGCGAACTT	60
	Primer 2	TCGCGGCGGCGGCGTCGG	CCGCTCGAGCTAGCGACACCCACAACCCTCCA	60
4A > C	Primer 1	CCGGAATTCACCATGGTGGCCGGGACC	CTGGTGTCCAAAAGTCTGGTCACGGG	60
	Primer 2	CCCCGTGACCAGACTTTTGGACACC	CCGCTCGAGCTAGCGACACCCACAACCCTCCA	60

### Cell culture

Mouse embryonic mesenchymal stem cell line (C3H10T1/2 cells) was obtained from the American Type Culture Collection (ATCC, Maryland Rockefeller,USA). The cells were cultured in basal medium eagle (BME, Gibco BRL, Grand Island, NY, USA) medium supplemented with 10% fetal bovine serum (FBS, Gibco BRL, Grand Island, NY, USA). For osteogenic differentiation, cells were seeded at a density of 4.5 × 10^5^ cells/well in 6-well plates and maintained in BME containing 10% FBS. Cells at 80% confluence were induced by osteogenic medium containing 100 nM dexamethasone (Sigma), 10 mM β-glycerophosphate (Sigma) and 0.2 mM ascorbic acid (Sigma) for 7–21 days. The culture medium was changed every 3 days and cells were grown at 37 °C in a humidified atmosphere containing 5% CO_2_.

### Transfection and stable selection

C3H10T1/2 cells were transfected with empty pcDNA3.1 (+) vector, pcDNA3.1/BMPR-IA (WT) vector, pcDNA3.1/BMPR-IA (MT -349C > T) vector, pcDNA3.1/BMPR-IA (MT 4A > C) vector, and pcDNA3.1/BMPR-IA (MT -349C > T and 4A > C) vector, respectively. For stable transfection, cells were seeded at 4.5 × 10^5^ cells/well in 6-well plates pre-coated with poly-l-lysine (Sigma, St Louis, MO, USA) and maintained in BME containing 10% FBS. After cells were grown to confluence, the gene transfections were performed using lipofectamine (Invitrogen) according to the manufacturer’s instructions. 4–6 h post-transfection, the transfected cells were cultured in osteogenic differentiation conditions with 200 ng/mL recombinant human BMP2 (rhBMP2, BioSia, Co., Ltd., Shanghai, China) for 72 h. The following day, the cells were trypsinized (Gibco) and plated into a large cell culture dish. Stably transfected C3H10T1/2 cells were selected with 800 μg/mL G418 media for 2 weeks and subsequently maintained at 200 μg/mL G418 media (Gibco), following which, G418-resistant clones were selected and expanded. After 6 weeks of G418 selection, the stably transfected cells were collected for subsequent experiments.

### Western blotting

Lysates from stably transfected C3H10T1/2 cells were obtained using the total protein extraction kit (Applygen Technologies Inc., Beijing, China). The concentrations of protein were measured using the bicinchoninic acid (BCA) assay and quantified using bovine serum albumin (BSA) protein standards according to the manufacturer’s instructions (Pierce, Rockford, IL, USA). The whole cell protein extracts were resolved by SDS-PAGE and transferred to nitrocellulose membrane. Pre-stained protein standards were used for determining the relative molecular mass of the proteins. The membranes were blocked in 5% nonfat dry milk in Tris-buffered saline, followed by incubation with the BMPR-IA (1:1000, Santa Cruz, CA, USA) or GAPDH monoclonal antibodies (1:1000, Santa Cruz) at 4 °C overnight, followed by incubation with a mouse anti-human HRP-conjugated secondary antibody (1:2000, Zhongshan Jinqiao, Beijing, China) for 1 h at room temperature. The protein levels of Smad4 and phosphorylated Smad1/5/8 were detected using specific monoclonal antibodies (1:000, Cell Signaling Technology; CST, Danvers, MA, USA), respectively, or GAPDH monoclonal antibody (1:1000, Santa Cruz) at 4 °C overnight, followed by 1 h incubation with a mouse anti-human HRP-conjugated secondary antibody (1:2000, Zhongshan Jinqiao, Beijing, China) at room temperature. The blots were washed and subjected to immuno-detection by chemiluminescence using SuperSignal (Pierce). The blots were scanned using the Kodak Image Station (440 CF; Kodak, Rochester, MN, USA), and the optical densities of the bands in each lane were analyzed to assess the variations in protein levels relative to GADPH.

### ALP activity assay

The ALP activity was detected using the alkaline phosphatase quantitative kit (Jiancheng, Nanjing, China). The stably transfected C3H10T1/2 cells were scraped off the culture dish using 50 mmol Tris-HCl and lysed by sonication. The sample tube (30 μL cell lysis solution, 0.5 mL buffer solution, and 0.5 mL matrix liquid), Standard tube (30 μL phenol standard solution 0.1 mg/mL, 0.5 mL buffer solution, and 0.5 mL matrix liquid), and blank tube (30 μL double-distilled water, 0.5 mL buffer solution, and 0.5 mL matrix liquid) were thoroughly mixed and incubated in a 37 °C water bath for 15 min, followed by the addition of 1.5 mL color reagent. The reaction mixtures were then agitated, and the absorbance measured at 520 nm. The ALP activity for each samples was measure by the following equation; the activity of ALP (U/gprot) = (determination tube absorbance/standard tube absorbance) × standard tube containing phenol (0.003 mg)/number of proteins in the sample.

### OC activity assay

The OC activity was detected using the Osteocalcin Radioimmunoassay Kit (Dongya, Beijing, China) according to the principle of the competition of radiation immunity. The stably transfected C3H10T1/2 cells were scraped off the cell culture plate using 50 mmol Tris-HCl and lysed by sonication. ^125^I- labeled OC and OC antibody were mixed for 24 h, followed by precipitation using the separation agent. After centrifugation at 4 °C, the radiation dose of the precipitate was detected using a γ-scintillation counter. The OC content of the sample was obtained based on the standard curve and presented as ng/mL.

### Statistical analysis

The Pearson Chi-square test was performed to assess the difference in the genotypic and allelic distribution between OPLL patients and control subjects. The Student unpaired t-test was used to compare the number of ossified cervical vertebrae between the two groups. A *p*-value < 0.05 was considered to be statistically significant (two-sided). ANOVA followed by Bonferroni post hoc multiple comparison was used to examine significant differences among the experimental groups. A adjust *p*-value< 0.003 was considered to be significant [24]. All data was expressed as means ± standard deviation and analyzed with SPSS 13.0 software (SPSS, Chicago, IL).

## Results

### Typing and analysis of SNP loci in the BMPR-IA gene

**Fig. 1:**
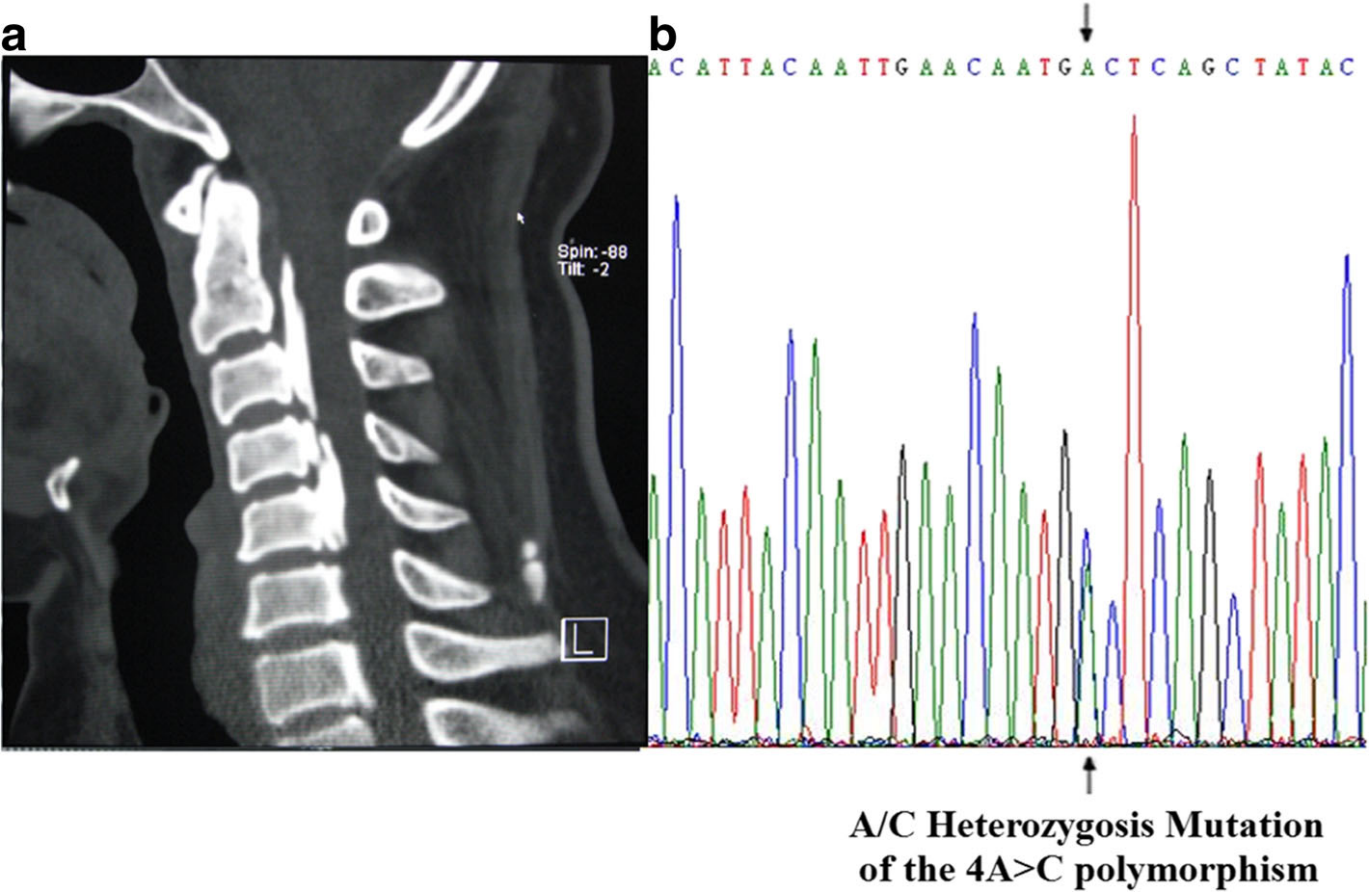
The 2-dimensional (2D) CT and sequencing results of a 43 year old woman with OPLL in the cervical spine. **a** The 2D CT showed that the patient had OPLL (C2–C3, C4-C5, C6, and C7 mixed type). **b** The direct sequencing results of the PCR products showing the A/C heterozygous mutation of 4A > C polymorphism in B*MPR-IA* gene. A allele: green line; T allele: red line; C allele: blue line; G allele: black line

**Fig. 2:**
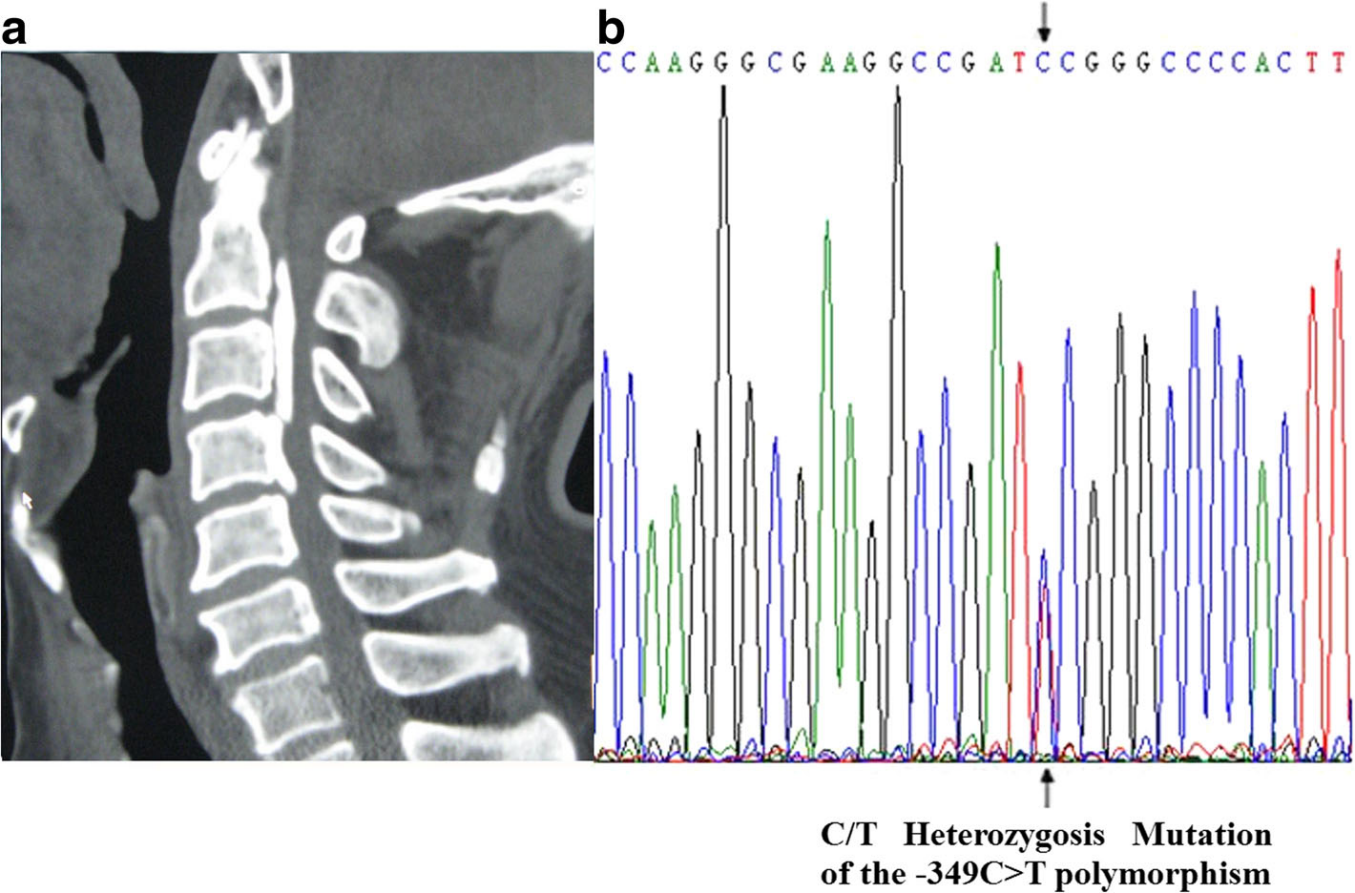
The 2-dimensional (2D) CT and sequencing results of a 68 year old man with OPLL in the cervical spine. **a** The 2D CT showed the patient had OPLL (C2–C3, C4, C5, and C6, mixed type). **b**The direct sequencing results of the PCR products showing the C/T heterozygous mutation of -349C > T polymorphism in *BMPR-IA* gene. A allele: green line; T allele: red line; C allele: blue line; G allele: black line

**Fig. 3:**
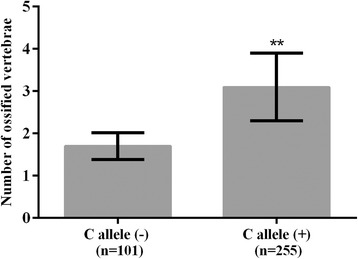
The distribution of ossified cervical vertebrae of OPLL patients in each subgroup classified by C allele type in 4A > C polymorphism of *BMPR-IA* gene. The number of ossified vertebrae is shown under each bar. Data is expressed as means (bars) ± SD (error bars). The difference in the number of ossified vertebrae between the non-carriers and carriers of C allele type was statistically significant. ** *P* < 0.001

The SNPs in the coding regions of *BMPR-IA* gene were detected by direct DNA sequencing (Figs. 1 and 2). The four SNPs analyzed in the exon regions of *BMPR-IA* gene included -349C > T in exon 1, the 4A > C in exon 3, the 1327C > T in exon 11, and the 1395G > C in exon 12. The genotypic and allelic distributions of the four SNP loci in *BMPR-IA* gene between OPLL patients and non-OPLL controls are shown in Table 3. The results showed that the -349C > T and the 4A > C polymorphisms in the coding region of *BMPR-IA* gene were significantly associated with the development of OPLL in the cervical spine. We observed that there was a significant difference between the 4A > C polymorphism in exon 3 of *BMPR-IA* gene and the occurrence and the extent of OPLL in the cervical spine (*P* < 0.001). The C allele type in 4A > C polymorphism significantly increased the occurrence and the extent of OPLL in the cervical spine (Fig. 3). In addition, there was a significant allelic frequency difference between the -349C > T polymorphism in exon 1 of *BMPR-IA* gene and the susceptibility to OPLL (*P* < 0.001), but did not affect the extent of OPLL in the cervical spine. The T allele type in -349C > T polymorphism significantly increased the susceptibility to OPLL, but not the extent of OPLL in the cervical spine.

**Table 3:** Genotypic and allelic distribution of four SNP loci in the exon regions of *BMPR-IA* gene between OPLL patients and non-OPLL Controls

SNPs	-349C > T			4A > C			1327C > T			1395G > C		
	exon1			exon3, Pro2Thr			exon11, Arg443Cys			exon12, Pro465Pro		
Genotype	CC	CT	TT	AA	AC	CC	CC	CT	TT	GG	GC	CC
OPLL (*n* = 356)	83	190	83	145	143	68	206	85	65	120	156	80
Control (*n* = 617)	297	246	74	283	284	50	370	148	99	240	222	155
Pearson Chi-Square Test	*P* < 0.001			*P* < 0.001			*P* = 0.662			*P* = 0.436		
Allele	C	T		A	C		C	T		G	C	
OPLL	356	356		433	279		497	215		396	316	
Control	840	394		850	384		888	346		702	532	
Pearson Chi-Square Test	*P* < 0.001			*P* < 0.001			*P* = 0.311			*P* = 0.586

### Construction of the *BMPR-IA* gene expression plasmids

**Fig. 4:**
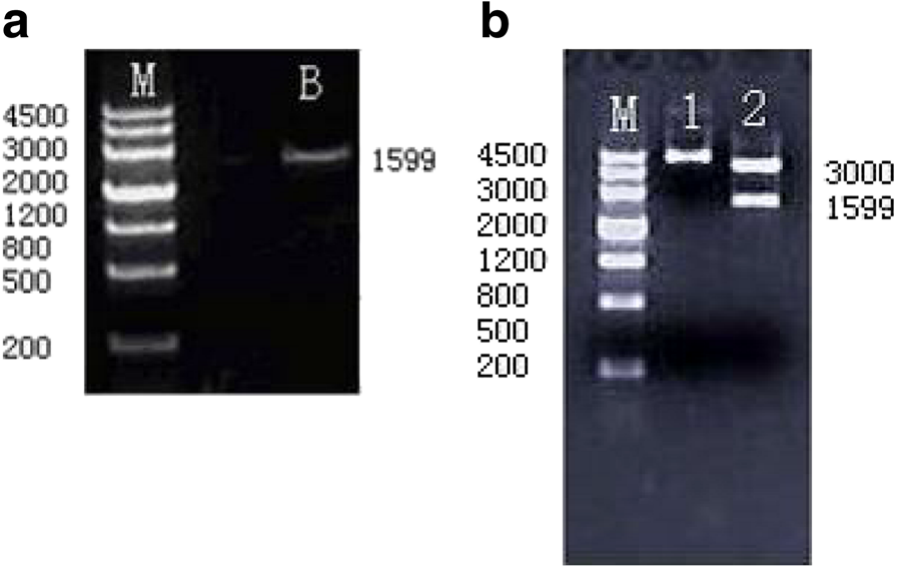
The *BMPR-IA* gene coding sequence and pGEM-T/BMPR-IA (WT) vector digested with Hind III/Bam HI enzyme were analyzed by 4% agarose gel electrophoresis, respectively. **a** The annealed ds-oligos of *BMPR-IA* gene coding sequence was analyzed by 4% agarose gel electrophoresis. Each ds oligo annealing reactant showed a detectable molecular weight band around 1599 bp, as expected for the length of the designed ds-oligos of *BMPR-IA* gene. Lane M: Marker, Lane B: *BMPR-IA* gene.0020**b** After the pGEM-T/BMPR-IA (WT) vector had been digested with Hind III/Bam HI enzyme, the fragments for *BMPR-IA* cDNA (1599 bp) and the pGEM-T vector (3000 bp) were observed in all the PCR products as analyzed by 4% agarose gel electrophoresis, respectively. The results showed that the ds-oligos of the *BMPR-IA* gene cDNA were ligated into the pGEM-T vector. Lane M: Marker, Lane 1: pGEM-T/BMPR-IA (WT) vector, Lane 2: The pGEM-T vector and *BMPR-IA* cDNA

The annealing efficiency of the ss oligos was analyzed by 4% agarose gel electrophoresis (Fig. 4a). The expected size of the ds oligos was 1599 bp. Each pair of ss oligos of the BMPR-IA gene encoding sequence was effectively annealed to form ds oligos, which were then ligated to linearized pGEM-T vector. The recombinant plasmid was then validated by digesting with Hind III-Bam H I enzyme. This generated two fragments, the *BMPR-IA* gene coding sequence (1599 bp) and the pGEM-T vector (3000 bp) (Fig. 4b). The BMPR-IA cDNA (1599 bp) was inserted into the pcDNA3.1 vector using T4 DNA ligase and transformed into DH5α. Analysis of the positive clones confirmed the orientation and sequence of the inserted ds-oligos.

### *BMPR-IA* gene expression in transfected C3H10T1/2 cells

**Fig. 5:**
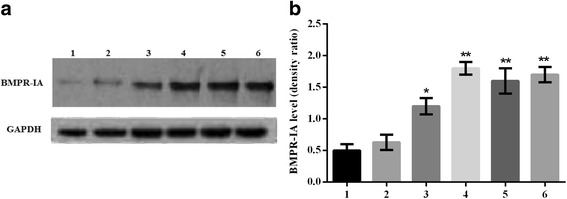
The expression of *BMPR-IA* gene in the transfected C3H10T1/2 cells was detected by Western blotting. **a** Western blot analysis of *BMPR-IA* protein levels in transfected C3H10T1/2 cells. Lane 1: C3H10T1/2 cells; Lane 2: C3H10T1/2/pcDNA3.1 cells; Lane 3: C3H10T1/2/pcDNA3.1/BMPR-IA (WT) cell; Lane 4 C3H10T1/2/pcDNA3.1/BMPR-IA (MT -349C > T) cells; Lane 5 C3H10T1/2/pcDNA3.1/BMPR-IA (MT 4A > C) cells; Lane 6 C3H10T1/2/pcDNA3.1/BMPR-IA (MT -349C > T and 4A > C) cells. **b** Statistical analysis of *BMPR-IA* protein expression levels in transfected C3H10T1/2 cells using the linear density ratio of BMPR-IA/GAPDH. The protein expression levels of *BMPR-IA* gene were significantly increased in C3H10T1/2/pcDNA3.1/BMPRIA (MT -349C > T, MT 4A > C, MT -349C > T and 4A > C) cells compared to the C3H10T1/2/pcDNA3.1/BMPRIA (WT) cells, C3H10T1/2/pcDNA3.1 cells, and C3H10T1/2 cells. *n* = 5, *p < 0.001 C3H10T1/2/pcDNA3.1/BMPRIA WT cells compared with C3H10T1/2 cells, *p < 0.001 C3H10T1/2/pcDNA3.1/BMPRIA WT cells compared with C3H10T1/2/pcDNA3.1 cells; **p < 0.001 C3H10T1/2/pcDNA3.1/BMPRIA MT -349C > T cells compared with C3H10T1/2 cells, **p < 0.001 C3H10T1/2/pcDNA3.1/BMPRIA MT -349C > T cells compared with C3H10T1/2/pcDNA3.1 cells; **p < 0.001 C3H10T1/2/pcDNA3.1/BMPRIA MT 4A > C cells compared with C3H10T1/2 cells, **p < 0.001 C3H10T1/2/pcDNA3.1/BMPRIA MT 4A > C cells compared with C3H10T1/2/pcDNA3.1 cells; **p < 0.001 C3H10T1/2/pcDNA3.1/BMPRIA MT -349C > T and 4A > C cells compared with C3H10T1/2 cells, **p < 0.001 C3H10T1/2/pcDNA3.1/BMPRIA MT -349C > T and 4A > C cells compared with C3H10T1/2/pcDNA3.1 cells; **p < 0.001 C3H10T1/2/pcDNA3.1/BMPRIA MT -349C > T cells compared with C3H10T1/2/pcDNA3.1/BMPRIA WT cells, **p < 0.001 C3H10T1/2/pcDNA3.1/BMPRIA MT 4A > C cells compared with C3H10T1/2/pcDNA3.1/BMPRIA WT cells, **p < 0.001 C3H10T1/2/pcDNA3.1/BMPRIA MT -349C > T and 4A > C cells compared with C3H10T1/2/pcDNA3.1/BMPRIA WT cells

The expression of *BMPR-IA* gene in the transfected C3H10T1/2 cells was determined by Western blotting (Fig. 5). The results demonstrated that the BMPR-IA protein levels were significantly increased in pcDNA3.1/BMPR-IA (WT, MT -349C > T, MT 4A > C, MT -349C > T and 4A > C) vector-transfected C3H10T1/2 cells compared to the control cells and empty pcDNA3.1 (+) vector-transfected cells (*n* = 5, *p < 0.001 C3H10T1/2/pcDNA3.1/BMPRIA WT cells compared with C3H10T1/2 cells, *p < 0.001 C3H10T1/2/pcDNA3.1/BMPRIA WT cells compared with C3H10T1/2/pcDNA3.1 cells; **p < 0.001 C3H10T1/2/pcDNA3.1/BMPRIA MT -349C > T cells compared with C3H10T1/2 cells, **p < 0.001 C3H10T1/2/pcDNA3.1/BMPRIA MT -349C > T cells compared with C3H10T1/2/pcDNA3.1 cells; **p < 0.001 C3H10T1/2/pcDNA3.1/BMPRIA MT 4A > C cells compared with C3H10T1/2 cells, **p < 0.001 C3H10T1/2/pcDNA3.1/BMPRIA MT 4A > C cells compared with C3H10T1/2/pcDNA3.1 cells; **p < 0.001 C3H10T1/2/pcDNA3.1/BMPRIA MT -349C > T and 4A > C cells compared with C3H10T1/2 cells, **p < 0.001 C3H10T1/2/pcDNA3.1/BMPRIA MT -349C > T and 4A > C cells compared with C3H10T1/2/pcDNA3.1 cells). This demonstrated that pcDNA3.1/BMPR-IA (WT, MT) vectors were successfully transfected into C3H10T1/2 cells and stably expressed. In addition, the BMPR-IA protein levels in pcDNA3.1/BMPR-IA (MT -349C > T, MT 4A > C, MT -349C > T and 4A > C) vector-transfected C3H10T1/2 cells were higher than in pcDNA3.1/BMPR-IA (WT) vector-transfected C3H10T1/2 cells (*n* = 5, **p < 0.001 C3H10T1/2/pcDNA3.1/BMPRIA MT -349C > T cells compared with C3H10T1/2/pcDNA3.1/BMPRIA WT cells, **p < 0.001 C3H10T1/2/pcDNA3.1/BMPRIA MT 4A > C cells compared with C3H10T1/2/pcDNA3.1/BMPRIA WT cells, **p < 0.001 C3H10T1/2/pcDNA3.1/BMPRIA MT -349C > T and 4A > C cells compared with C3H10T1/2/pcDNA3.1/BMPRIA WT cells).

### Phosphorylated Smad1/5/8 and Smad4 expression in transfected C3H10T1/2 cells

**Fig. 6:**
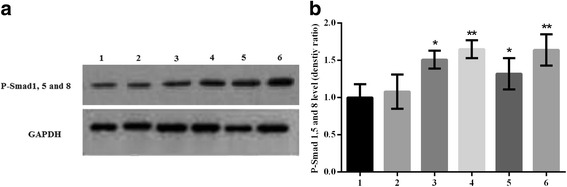
The proteins levels of phosphorylated Smad1/5/8 in the transfected C3H10T1/2 cells were detected by Western blotting. **a** Western blot analysis of phosphorylated Smad1/5/8 proteins levels in transfected C3H10T1/2 cells. Lane 1 C3H10T1/2 cells; Lane 2 C3H10T1/2/pcDNA3.1 cells; Lane 3 C3H10T1/2/pcDNA3.1/BMPRIA (WT) cells; Lane 4 C3H10T1/2/pcDNA3.1/BMPR-IA (MT -349C > T) cells; Lane 5 C3H10T1/2/pcDNA3.1/BMPR-IA (MT 4A > C) cells; Lane 6 C3H10T1/2/pcDNA3.1/BMPR-IA (MT -349C > T and 4A > C) cells. **b** Statistical analysis of phosphorylated Smad1/5/8 proteins levels in C3H10T1/2 cells, C3H10T1/2/pcDNA3.1 cells, C3H10T1/2/pcDNA3.1/BMPR-IA (WT) cells, and C3H10T1/2/pcDNA3.1/BMPR-IA (MT -349C > T, MT 4A > C, MT -349C > T and 4A > C) cells using the linear density ratio of phosphorylated Smad1/5/8/GAPDH. The phosphorylated Smad1/5/8 proteins levels were significantly increased in C3H10T1/2/pcDNA3.1/BMPR-IA (MT -349C > T, MT -349C > T and 4A > C) cells compared to C3H10T1/2/pcDNA3.1/BMPR-IA (WT) cells, C3H10T1/2/pcDNA3.1 cells, and C3H10T1/2 cells. *n* = 5, *p < 0.001 C3H10T1/2/pcDNA3.1/BMPRIA WT cells compared with C3H10T1/2 cells, *p < 0.001 C3H10T1/2/pcDNA3.1/BMPRIA WT cells compared with C3H10T1/2/pcDNA3.1 cells; **p < 0.001 C3H10T1/2/pcDNA3.1/BMPRIA MT -349C > T cells compared with C3H10T1/2 cells, **p < 0.001 C3H10T1/2/pcDNA3.1/BMPRIA MT -349C > T cells compared with C3H10T1/2/pcDNA3.1 cells; *p < 0.001 C3H10T1/2/pcDNA3.1/BMPRIA MT 4A > C cells compared with C3H10T1/2 cells, *p < 0.001 C3H10T1/2/pcDNA3.1/BMPRIA MT 4A > C cells compared with C3H10T1/2/pcDNA3.1 cells; **p < 0.001 C3H10T1/2/pcDNA3.1/BMPRIA MT -349C > T cells compared with C3H10T1/2 cells, **p < 0.001 C3H10T1/2/pcDNA3.1/BMPRIA MT -349C > T and 4A > C cells compared with C3H10T1/2/pcDNA3.1 cells; **p < 0.001 C3H10T1/2/pcDNA3.1/BMPRIA MT -349C > T cells compared with C3H10T1/2/pcDNA3.1/BMPRIA WT cells, **p < 0.001 C3H10T1/2/pcDNA3.1/BMPRIA MT -349C > T and 4A > C cells compared with C3H10T1/2/pcDNA3.1/BMPRIA WT cells. The protein levels of phosphorylated Smad1/5/8 were not increased significantly in C3H10T1/2/pcDNA3.1/BMPR-IA (MT 4A > C) cells compared to the C3H10T1/2/pcDNA3.1/BMPR-IA (WT) cells (*n* = 5)

**Fig. 7:**
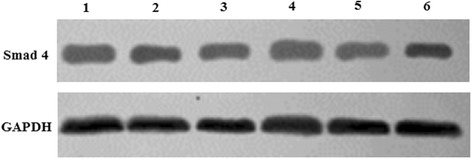
Western blot analysis of Smad4 protein levels in the transfected C3H10T1/2 cells. Lane 1 C3H10T1/2 cells; Lane 2 C3H10T1/2/pcDNA3.1 cells; Lane 3 C3H10T1/2/pcDNA3.1/BMPR-IA (WT) cells; Lane 4 C3H10T1/2/pcDNA3.1/BMPR-IA (MT -349C > T) cells; Lane 5 C3H10T1/2/pcDNA3.1/BMPR-IA (MT 4A > C) cells; Lane 6 C3H10T1/2/pcDNA3.1/BMPR-IA (MT -349C > T and 4A > C) cells. There was no significant differences in Smad4 protein levels among the experimental groups (*n* = 5)

The proteins levels of Smad4 and phosphorylated Smad1/5/8 were determined by Western blotting (Figs. 6, 7). The phosphorylation levels of Smad1/5/8 were increased significantly in pcDNA3.1/BMPR-IA (WT, MT -349C > T, MT 4A > C, MT -349C > T and 4A > C) vector-transfected C3H10T1/2 cells compared to control C3H10T1/2 cells and empty pcDNA3.1 (+) vector-transfected C3H10T1/2 cells (*n* = 5, *p < 0.001 C3H10T1/2/pcDNA3.1/BMPRIA WT cells compared with C3H10T1/2 cells, *p < 0.001 C3H10T1/2/pcDNA3.1/BMPRIA WT cells compared with C3H10T1/2/pcDNA3.1 cells; **p < 0.001 C3H10T1/2/pcDNA3.1/BMPRIA MT -349C > T cells compared with C3H10T1/2 cells, **p < 0.001 C3H10T1/2/pcDNA3.1/BMPRIA MT -349C > T cells compared with C3H10T1/2/pcDNA3.1 cells; *p < 0.001 C3H10T1/2/pcDNA3.1/BMPRIA MT 4A > C cells compared with C3H10T1/2 cells, *p < 0.001 C3H10T1/2/pcDNA3.1/BMPRIA MT 4A > C cells compared with C3H10T1/2/pcDNA3.1 cells; **p < 0.001 C3H10T1/2/pcDNA3.1/BMPRIA MT -349C > T cells compared with C3H10T1/2 cells, **p < 0.001 C3H10T1/2/pcDNA3.1/BMPRIA MT -349C > T and 4A > C cells compared with C3H10T1/2/pcDNA3.1 cells). In addition, the phosphorylation levels of Smad1/5/8 were significantly increased in pcDNA3.1/BMPR-IA (MT -349C > T, MT -349C > T and 4A > C) vector-transfected C3H10T1/2 cells compared to pcDNA3.1/BMPR-IA (WT) vector-transfected C3H10T1/2 cells (*n* = 5, **p < 0.001 C3H10T1/2/pcDNA3.1/BMPRIA MT -349C > T cells compared with C3H10T1/2/pcDNA3.1/BMPRIA WT cells, **p < 0.001 C3H10T1/2/pcDNA3.1/BMPRIA MT -349C > T and 4A > C cells compared with C3H10T1/2/pcDNA3.1/BMPRIA WT cells). However, no significant differences were observed in the phosphorylation levels of Smad1/5/8 between pcDNA3.1/BMPR-IA (MT 4A > C) and pcDNA3.1/BMPR-IA (WT) vector-transfected cells (n = 5). The Smad4 protein levels also did not differ significantly among the experimental groups (*n* = 5).

### ALP and OC activity in transfected C3H10T1/2 cells

**Fig. 8:**
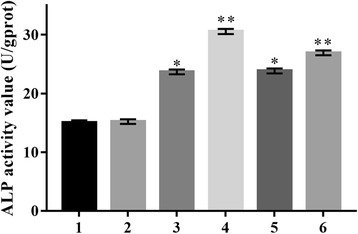
The ALP activity in transfected C3H10T1/2 cells. The ALP activity levels were significantly increased in C3H10T1/2/pcDNA3.1/BMPR-IA (MT -349C > T 30.56 ± 0.44 U/gprot, MT -349C > T and 4A > C 26.91 ± 0.41 U/gprot) cells compared to C3H10T1/2/pcDNA3.1/BMPR-IA (WT 23.67 ± 0.42 U/gprot) cells. n = 5, *p < 0.001 C3H10T1/2/pcDNA3.1/BMPRIA WT cells compared with C3H10T1/2 cells, *p < 0.001 C3H10T1/2/pcDNA3.1/BMPRIA WT cells compared with C3H10T1/2/pcDNA3.1 cells; **p < 0.001 C3H10T1/2/pcDNA3.1/BMPRIA MT -349C > T cells compared with C3H10T1/2 cells, **p < 0.001 C3H10T1/2/pcDNA3.1/BMPRIA MT -349C > T cells compared with C3H10T1/2/pcDNA3.1 cells; *p < 0.001 C3H10T1/2/pcDNA3.1/BMPRIA MT 4A > C cells compared with C3H10T1/2 cells, *p < 0.001 C3H10T1/2/pcDNA3.1/BMPRIA MT 4A > C cells compared with C3H10T1/2/pcDNA3.1 cells; **p < 0.001 C3H10T1/2/pcDNA3.1/BMPRIA MT -349C > T and 4A > C cells compared with C3H10T1/2 cells, **p < 0.001 C3H10T1/2/pcDNA3.1/BMPRIA MT -349C > T and 4A > C cells compared with C3H10T1/2/pcDNA3.1 cells; **p < 0.001 C3H10T1/2/pcDNA3.1/BMPRIA MT -349C > T cells compared with C3H10T1/2/pcDNA3.1/BMPRIA WT cells, **p < 0.001 C3H10T1/2/pcDNA3.1/BMPRIA MT -349C > T and 4A > C cells compared with C3H10T1/2/pcDNA3.1/BMPRIA WT cells. No differences in ALP activity in C3H10T1/2/pcDNA3.1/BMPR-IA (MT 4A > C 23.84 ± 0.42 U/gprot) cells were observed compared to the C3H10T1/2/pcDNA3.1/BMPR-IA (WT 23.67 ± 0.42 U/gprot) cells (*n* = 5)

**Fig. 9:**
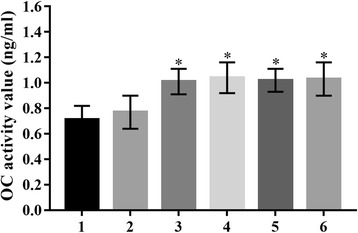
The OC activity in transfected C3H10T1/2 cells. The OC activity levels were significantly increased in C3H10T1/2/pcDNA3.1/BMPR-IA (WT 1.01 ± 0.10 ng/mL, MT -349C > T 1.04 ± 0.12 ng/mL, MT 4A > C 1.02 ± 0.09 ng/mL, MT -349C > T and 4A > C 1.03 ± 0.13 ng/mL) cells compared to the control groups. n = 5, *p < 0.001 C3H10T1/2/pcDNA3.1/BMPRIA WT cells compared with C3H10T1/2 cells, *p < 0.001 C3H10T1/2/pcDNA3.1/BMPRIA WT cells compared with C3H10T1/2/pcDNA3.1 cells; *p < 0.001 C3H10T1/2/pcDNA3.1/BMPRIA MT -349C > T cells compared with C3H10T1/2 cells, *p < 0.001 C3H10T1/2/pcDNA3.1/BMPRIA MT -349C > T cells compared with C3H10T1/2/pcDNA3.1 cells; *p < 0.001 C3H10T1/2/pcDNA3.1/BMPRIA MT 4A > C cells compared with C3H10T1/2 cells, *p < 0.001 C3H10T1/2/pcDNA3.1/BMPRIA MT 4A > C cells compared with C3H10T1/2/pcDNA3.1 cells; *p < 0.001 C3H10T1/2/pcDNA3.1/BMPRIA MT -349C > T and 4A > C cells compared with C3H10T1/2 cells, *p < 0.001 C3H10T1/2/pcDNA3.1/BMPRIA MT -349C > T and 4A > C cells compared with C3H10T1/2/pcDNA3.1 cells. However, no significant differences were observed in OC activity between C3H10T1/2/pcDNA3.1/BMPRIA (WT 1.01 ± 0.10 ng/ml) cells and C3H10T1/2/pcDNA3.1/BMPRIA (MT -349C > T 1.04 ± 0.12 ng/mL, MT 4A > C 1.02 ± 0.09 ng/mL, MT -349C > T and 4A > C 1.03 ± 0.13 ng/mL) cells (n = 5)

The activities of ALP and OC were determined in stably transfected C3H10T1/2 cells by ALP quantitation and osteocalcin radioimmunoassay kits, respectively (Figs. 8, 9). The ALP activity was increased in pcDNA3.1/BMPR-IA (WT 23.67 ± 0.42 U/gprot, MT -349C > T 30.56 ± 0.44 U/gprot, MT 4A > C 23.84 ± 0.42 U/gprot, MT -349C > T and 4A > C 26.91 ± 0.41 U/gprot) vector-transfected C3H10T1/2 cells compared to control C3H10T1/2 cells (14.74 ± 0.46 U/gprot) and empty pcDNA3.1 (+) vector-transfected C3H10T1/2 cells (15.08 ± 0.51 U/gprot) (*n* = 5). ALP activity was significantly higher in pcDNA3.1/BMPR-IA (MT -349C > T 30.56 ± 0.44 U/gprot, MT -349C > T and 4A > C 29.91 ± 0.41 U/gprot) vectors-transfected C3H10T1/2 cells compared to pcDNA3.1/BMPR-IA (WT 23.67 ± 0.42 U/gprot) vector-transfected C3H10T1/2 cells (*n* = 5). However, no significant differences were observed in the ALP activity between pcDNA3.1/BMPR-IA (MT 4A > C 23.84 ± 0.42 U/gprot) and pcDNA3.1/BMPR-IA (WT 23.67 ± 0.42 U/gprot) vector-transfected C3H10T1/2 cells (*n* = 5). In addition, the OC activity increased in pcDNA3.1/BMPRIA (WT 1.01 ± 0.10 ng/mL, MT -349C > T 1.04 ± 0.12 ng/mL, MT 4A > C 1.02 ± 0.09 ng/mL, MT -349C > T and 4A > C 1.03 ± 0.13 ng/ml) vector-transfected C3H10T1/2 cells compared to the control C3H10T1/2 (0.72 ± 0.11 ng/mL) cells and empty pcDNA3.1 (+) (0.79 ± 0.12 ng/mL) vector-transfected C3H10T1/2 cells (n = 5). In addition, no significant differences in the OC activity were observed between pcDNA3.1/BMPRIA (WT 1.01 ± 0.10 ng/mL) vector-transfected C3H10T1/2 cells and pcDNA3.1/BMPRIA (MT -349C > T 1.04 ± 0.12 ng/mL, MT 4A > C 1.02 ± 0.09 ng/mL, MT -349C > T and 4A > C 1.03 ± 0.13 ng/mL) vector-transfected C3H10T1/2 cells (n = 5).

## Discussion

OPLL of the spine is a pathological condition that causes ossification in the posterior longitudinal ligament of the spine with the formation of ectopic bone primarily through endochondral ossification [25, 26]. The ossification of the spinal ligament causes myelopathy, radiculopathy, or both and leads to severe neurological deficits and paralysis. Although the incidence of OPLL is higher in Asian countries than non-Asian [1], the pathogenesis is unclear. OPLL is a multifactorial disease and may result from genetic, gene interactions and various environmental factors. Epidemiological studies have demonstrated that genetic susceptibility may be a critical factor for the development of OPLL, and genetic components appear to play a crucial role in its pathogenesis [2–4]. Previous studies have indicated that SNPs in BMP2, 4, and 9, collagen 6A1, angiotensin I converting enzyme gene, BH3 interacting domain death agonist, nucleotide pyrophosphatase, and vitamin K epoxide reductase complex subunit 1 are associated with the development of OPLL. The occurrence and development of OPLL are regulated by various factors and several genes [4–9]. However, identifying all the susceptible genes for OPLL, especially the molecular machinery underlying these genetic polymorphisms is challenging.

BMPRs are the receptor binding proteins of BMPs, and include type I and II serine/threonine kinase receptors. Receptor-ligand BMPs are involved in the formation of new bone and cartilage, and play a vital role in bone formation during different developmental stages. The binding of BMP to at least one type I and one type II serine/threonine kinase receptor is essential for the activation of BMP signaling [27]. BMPs exert their effects via hetero-oligomeric complexes of type I and II receptors. There are three type I receptors: BMPR-IA, BMPR-IB, and ActR-IA and three type II receptors: BMPR- II, ActR-II, and ActR-IIB. BMPR-IA, IB, and II are specific for BMPs, as well as for ActR-IA, II, and IIB, which are also signaling receptors for activins [10]. BMP bind with different affinities to specific type I and II receptors. The type I BMP receptors have been reported as the high-affinity binding receptors, whereas type II BMP receptors bind BMPs directly with low affinity [11]. The affinity of the BMPs to receptors is crucial for signal transduction activity [28]. The type I BMP receptors play a central role in BMP signal transduction from the receptor to target genes in the nucleus.

The *BMPR-IA* gene on chromosome 10 is a subtype of type I BMP receptors and contains 13 exons. Previous studies have suggested that the levels of protein and mRNA of *BMPR-IA* gene were highly expressed in chondrocytes of fibrocartilage tissue around the calcified zone and fibroblast-like spindle cells in non-ossified ligaments of OPLL patients. However, the gene was not expressed in ligament tissues in non-OPLL patients [18, 20]. Our present study demonstrates that BMPR-IA, which transduces signals for BMPs, plays an important role in the pathological ossification of OPLL.

Previously, we reported that the 4A > C and the -349C > T polymorphisms of *BMPR-IA* gene was associated with OPLL in the cervical spine [21]. In this study, we further validated our findings using a larger cohort. The C allele type in the 4A > C polymorphism was significantly associated with the occurrence and extent of OPLL in the cervical spine. The T allele type in the -349C > T polymorphism increased the susceptibility to OPLL of the cervical spine but not the extent. However, the molecular mechanisms underlying the two SNPs in the *BMPR-IA* gene have not yet been elucidated. Based on our preliminary data, a series of in vitro experiments were performed to determine whether Smad signaling pathway may be involved in the increased susceptibility and severity to OPLL via these *BMPR-IA* gene polymorphisms.

Signal transduction studies have demonstrated that the immediate downstream molecules of BMP receptors are Smad proteins, especially Smad1, 5 and 8, which play a central role in BMP signal transduction. Binding of BMP ligand to at least one type I and one type II BMP receptors results in the type II BMP receptor phosphorylating the type I receptor [29], which subsequently leads to the recruitment of the receptor-activated Smads (R-Smads, Smads 1, 5 and 8). The activation of type I BMP receptor is required for the direct interaction between type I receptor and R-Smads (Smads 1, 5 and 8). R-Smads associate directly and transiently with activated type I BMP receptors and undergo direct phosphorylation at the C-terminal SSXS motif of Smad in a ligand-dependent manner [30, 31]. R-Smads are the only Smads that have an SSXS motif in their C-terminal region. After rapid release from the type I receptor, the phosphorylated R-Smads proteins form hetero-oligomeric complexes with the common-mediator Smads (Co-Smads, Smad4), which acts as a shared partner. The R-Smads and Co-Smad complexes then translocate into the nucleus to regulate the transcription of specific target genes with other transcription factors. In the nucleus, the Smads 1 and 5 proteins exert their transcriptional activity by direct interaction with DNA and association with other DNA-binding proteins [32]. ALP, OC, and type I collagen are osteogenesis-specific protein factors in the downstream regulation of the BMP signaling pathway. Moreover, ALP is essential for the osteogenic phenotype and is critical for bone formation mediated by the regulation of the mineralization of bone matrix [33, 34].

In order to determine the role of the -349C > T and 4A > C polymorphisms of *BMPR-IA* gene in the osteogenesis mechanism during the development of OPLL, we examined whether these two SNPs affected *BMPR-IA* gene expression and signal transduction of the Smad signaling pathway. The present study demonstrated that the expression levels of *BMPR-IA* gene were significantly increased in pcDNA3.1/BMPR-IA (MT -349C > T, MT 4A > C, MT -349C > T and 4A > C) vector-transfected C3H10T1/2 cells compared to the pcDNA3.1/BMPR-IA (WT) vector-transfected C3H10T1/2 cells. These data suggest that *BMPR-IA* gene may be overexpressed in pcDNA3.1/BMPR-IA (MT -349C > T, MT 4A > C, MT -349C > T and 4A > C) vector-transfected C3H10T1/2 cells. The phosphorylation levels of Smad1/5/8 were significantly increased in pcDNA3.1/BMPR-IA (MT -349C > T) vector-transfected C3H10T1/2 cells compared to the pcDNA3.1/BMPR-IA (WT) vector-transfected cells. This may indicate that the -349C > T polymorphism of *BMPR-IA* gene is positively associated with the phosphorylation of Smad1/5/8 expression levels. In addition, the ALP activity was significantly higher in the pcDNA3.1/BMPR-IA (MT -349C > T) vector-transfected C3H10T1/2 cells compared to the pcDNA3.1/BMPR-IA (WT) vector-transfected. These observations demonstrate that the -349C > T polymorphism of *BMPR-IA* gene is significantly associated with ALP activity, and suggest that the -349C > T polymorphism of *BMPR-IA* gene can enhance ALP activity via the Smad signaling pathway. Moreover, our results demonstrated that the overexpression of the *BMPR-IA* gene induced by the -349C > T polymorphism could increase the phosphorylation levels of Smad1/5/8, and further lead to the increase in ALP activity of osteogenesis-specific protein factors. Based on previous BMP receptor signal transduction studies, we propose that the -349C > T polymorphism of the BMPR-IA gene may play an important role in mediating susceptibility to OPLL via Smad signaling pathway. However, no significant differences were observed in the levels of Co-Smad4 protein among the experimental groups. There were no significant differences in the OC activity between pcDNA3.1/BMPRIA (WT) vector-transfected C3H10T1/2 cells and pcDNA3.1/BMPRIA (MT -349C > T, MT 4A > C, MT -349C > T and 4A > C) vector-transfected C3H10T1/2 cells. These results indicate that the -349C > T and 4A > C polymorphisms of BMPR-IA gene do not affect genetic predisposition to OPLL that is mediated through the increased levels of the Co-Smad4 protein and OC activity. In addition, we found that the protein levels of phosphorylated Smad1/5/8 and the ALP activity were not increased significantly in pcDNA3.1/BMPR-IA (MT 4A > C) vector-transfected C3H10T1/2 cells compared to the pcDNA3.1/BMPR-IA (WT) vector-transfected C3H10T1/2 cells. This result led us to hypothesize that the 4A > C polymorphism in the BMPR-IA gene may increase the susceptibility and severity of OPLL through another signaling pathway in addition to Smads. Based on previous studies, in addition to Smad signaling pathway, BMP binding to a homomeric type I or II BMPR leads to secondary formation of heterotetrameric complexes that activates non-Smad signaling pathways such as mitogen-activated protein kinase (MAPK) family of molecules including p38 and ERK1/2.

Finally, we would like to highlight some limitations in our study. We did not examine the protein levels of TAB1/TAK1, p38, and ERK1/2 in C3H10T1/2 cells and hence unable to state whether non-Smad signaling pathways are involved in the pathogenesis of OPLL. Future studies are necessary to examine if non-Smad signaling pathways are involved in the development of OPLL.

## Conclusions

The present results demonstrate that the expression levels of BMPR-IA gene, the levels of phosphorylated Smad1/5/8 and ALP activity were significantly increased in pcDNA3.1/BMPR-IA (MT -349C > T) vector-transfected C3H10T1/2 cells than the WT vector-transfected cells. Our data suggest that Smad signaling pathway may play important roles in the pathological process of OPLL induced by SNPs in BMPR-IA gene. The current study may help to clarify the molecular mechanisms underlying the susceptibility of the gene to OPLL, thereby providing a novel potential target for the diagnosis and therapy for OPLL.

## Abbreviations

ALP: Alkaline phosphatase
BME: Basal medium eagle
BMP: Bone morphogenetic protein
*BMPR-IA*: Bone morphogenetic protein receptor type IA
BMPRs: Bone morphogenetic protein receptors
C3H10T1/2: The mouse embryonic mesenchymal stem cells
DNA: Deoxyribonucleic acid
DS: Double-stranded
FBS: Fetal bovine serum
MT: Mutation type
OC: Osteocalcin
OPLL: Ossification of the posterior longitudinal ligament
PCR: Polymerase chain reaction
SNPs: Single nucleotide polymorphisms
SS: Single-stranded
WT: Wild type
